# Comparison of children and adults in deep brain stimulation for Tourette Syndrome: a large-scale multicenter study of 102 cases with long-term follow-up

**DOI:** 10.1186/s12916-024-03432-w

**Published:** 2024-05-30

**Authors:** Yuan Gao, Shu Wang, Anni Wang, Shiying Fan, Yan Ge, Huimin Wang, Dongmei Gao, Jian Wang, Zhiqi Mao, Hulin Zhao, Hua Zhang, Lin Shi, Huanguang Liu, Guanyu Zhu, Anchao Yang, Yutong Bai, Xin Zhang, Chong Liu, Qiao Wang, Renpeng Li, Kun Liang, Kayla Giovanna Brown, Zhiqiang Cui, Chunlei Han, Jianguo Zhang, Fangang Meng

**Affiliations:** 1 Beijing Neurosurgical Institute, Beijing Tiantan Hospital, Capital Medical University, Beijing, 100070 China; 2 Beijing Key Laboratory of Neurostimulation, Beijing, 100070 China; 3 Department of Neurosurgery, Beijing Tiantan Hospital, Capital Medical University, Beijing, 100070 China; 4 Department of Neurosurgery, Beijing Fengtai Hospital, Beijing, 100070 China; 5 Department of Neurosurgery, The First Medical Center of Chinese, PLA General Hospital, Beijing, 100853 China; 6 China Chinese Institute for Brain Research, Beijing, 102206 China

**Keywords:** Tourette syndrome, Neurodevelopmental disorders, Movement disorders, Deep brain stimulation, Pediatric surgery

## Abstract

**Background:**

Deep brain stimulation (DBS) is a promising therapy for refractory Gilles de la Tourette syndrome (GTS). However, its long-term efficacy, safety, and recommended surgical age remain controversial, requiring evidence to compare different age categories.

**Methods:**

This retrospective cohort study recruited 102 GTS patients who underwent DBS between October 2006 and April 2022 at two national centers. Patients were divided into two age categories: children (aged < 18 years; *n* = 34) and adults (aged ≥ 18 years; *n* = 68). The longitudinal outcomes as tic symptoms were assessed by the YGTSS, and the YBOCS, BDI, and GTS-QOL were evaluated for symptoms of obsessive–compulsive disorder (OCD), depression, and quality of life, respectively.

**Results:**

Overall, these included patients who finished a median 60-month follow-up, with no significant difference between children and adults (*p* = 0.44). Overall, the YGTSS total score showed significant postoperative improvements and further improved with time (improved 45.2%, 51.6%, 55.5%, 55.6%, 57.8%, 61.4% after 6, 12, 24, 36, 48, and ≥ 60 months of follow-up compared to baseline, respectively) in all included patients (all *p* < 0.05). A significantly higher improvement was revealed in children than adults at ≥ 60 months of follow-up in the YGTSS scores (70.1% vs 55.9%, *p* = 0.043), and the time to achieve 60% improvement was significantly shorter in the children group (median 6 months vs 12 months, *p* = 0.013). At the last follow-up, the mean improvements were 45.4%, 48.9%, and 55.9% and 40.3%, 45.4%, and 47.9% in YBOCS, BDI, and GTS-QOL scores for children and adults, respectively, which all significantly improved compared to baseline (all *p* < 0.05) but without significant differences between these two groups (all *p* > 0.05), and the children group received significantly higher improvement in GTS-QOL scores than adults (55.9% vs. 47.9%, *p* = 0.049).

**Conclusions:**

DBS showed acceptable long-term efficacy and safety for both children and adults with GTS. Surgeries performed for patients younger than 18 years seemed to show acceptable long-term efficacy and safety and were not associated with increased risks of loss of benefit compared to patients older than 18 at the time of surgery. However, surgeries for children should also be performed cautiously to ensure their refractoriness and safety.

**Supplementary Information:**

The online version contains supplementary material available at 10.1186/s12916-024-03432-w.

## Background

Gilles de la Tourette syndrome (GTS) is a chronic, complex neuropsychiatric disorder characterized by motor and phonic tics that typically begins in childhood between the ages of 6 and 8 years [1–3]. Approximately 50–90% of patients with GTS have comorbid psychiatric symptoms, which mainly include obsessive–compulsive disorder/compulsive behavior (OCD/OCB), depression, and other psychiatric and behavioral symptoms [2, 4]. The pathophysiology of GTS may be related to the cortico-striato-thalamo-cortical circuit and basal ganglia, but the exact mechanism remains unproven [5, 6]. Although medications and psycho-behavioral interventions are available for GTS patients, some of them still have severe persistent treatment-refractory motor and vocal tics, which did not respond to psycho-behavioral interventions per current expert standards and pharmacological treatments from three pharmacological classes (including alpha-adrenergic agonist, two dopamine antagonists, and a drug from at least one additional class) [7], influencing their quality of life and causing a heavy burden on patients, their families, and society [8, 9].

Deep brain stimulation (DBS) has been one of the most effective therapies for patients with movement disorders since it was first applied in the 1980s. It has since become much more popular for different neurological conditions [10–13]. In 1999, Vandewalle et al. first reported the use of DBS in treating refractory GTS patients [14]. Subsequently, the globus pallidus internus (GPi), thalamic centromedian nucleus (CM), and other targets of DBS have been proposed for GTS [14] and have shown promising efficacy [15–18], which may be related to the inhibition or functional override pathological network hyperactivity of cortico-striato-thalamo-cortical circuit [19]. In 2023, Rissardo et al. reviewed that only 11 studies about GTS related to DBS are registered on ClinicalTrials.gov [19]. Limited studies with small sample sizes are available on the long-term efficacy and safety of DBS for GTS, and one of the highly controversial questions is its optimal surgical age. Recommendations from the Tourette Syndrome Association in 2006 stated that TS patients should be over 25 years of age at surgery for DBS [20]. Nevertheless, with growing evidence on DBS for children and young adults, their updated recommendations in 2015 have removed the previously suggested 25-year-old age limit. However, they were still cautious in suggesting DBS for TS persons younger than 18 years of age should be consulted for ethical reasons and urgent indications [7]. This inconsistency relates to currently limited evidence on DBS for TS in the comparison of different age categories, especially for children and adults, in their efficacy and safety, which is a research gap that urgently needs to be filled.

In this large-scale multicenter study, we currently recruited the largest sample size of patients with GTS who underwent DBS at different surgical ages, especially including large national cohorts with great experience in both adult and pediatric surgeries (*n* = 102; children *n* = 34 and adults *n* = 68) with long-term follow-ups (a median 60-month follow-up) to compare the efficacy and safety of different age categories. This study provides substantial evidence on the long-term efficacy and safety of DBS surgeries for different age categories, especially in comparisons of children and adults, and might be a helpful reference for further understanding the recommended optimal surgical age of DBS for GTS.

## Methods

### Eligibility criteria and patients

This study is a retrospective longitudinal cohort study. Consecutive patients were recruited from two national centers, Beijing Tiantan Hospital, Capital Medical University, and the First Medical Center of Chinese PLA General Hospital, between October 2006 and April 2022. All these patients received GPi-DBS to treat, refractory GTS. All included participants, or their guardians signed written informed consent for the surgery performed and permissions for the anonymous scientific use of their data. This study was approved by the ethical committees of Beijing Tiantan Hospital, Capital Medical University (KY2021-159–01), and the First Medical Center of Chinese PLA General Hospital (S2019-268–02) and was conducted following the 1964 Helsinki Declaration and its later amendments.

The inclusion criteria were as follows: (1) patients with clinical diagnoses of GTS according to the Diagnostic and Statistical Manual of Mental Disorders Fifth Edition (DSM-V) by qualified neurologists and psychiatrists; (2) severe treatment-refractory symptoms, defined as symptoms that seriously impair the quality of life and might even be life-threatening, which could not be effectively controlled by at least two well-established medications and psycho-behavioral interventions in adequate doses and frequency; (3) received DBS treatment between October 2006 and April 2022; and (4) finished at least 6 months of follow-up. The exclusion criteria were as follows: (1) patients with medical conditions as surgical contraindications, such as severe cardiovascular diseases, pulmonary diseases, and others; or (2) patients with no controlled severe psychotic disorders (such as schizophrenia), which may influence surgical counseling, evaluation, nursing, postoperative rehabilitation, and related evaluations.

**Fig. 1:**
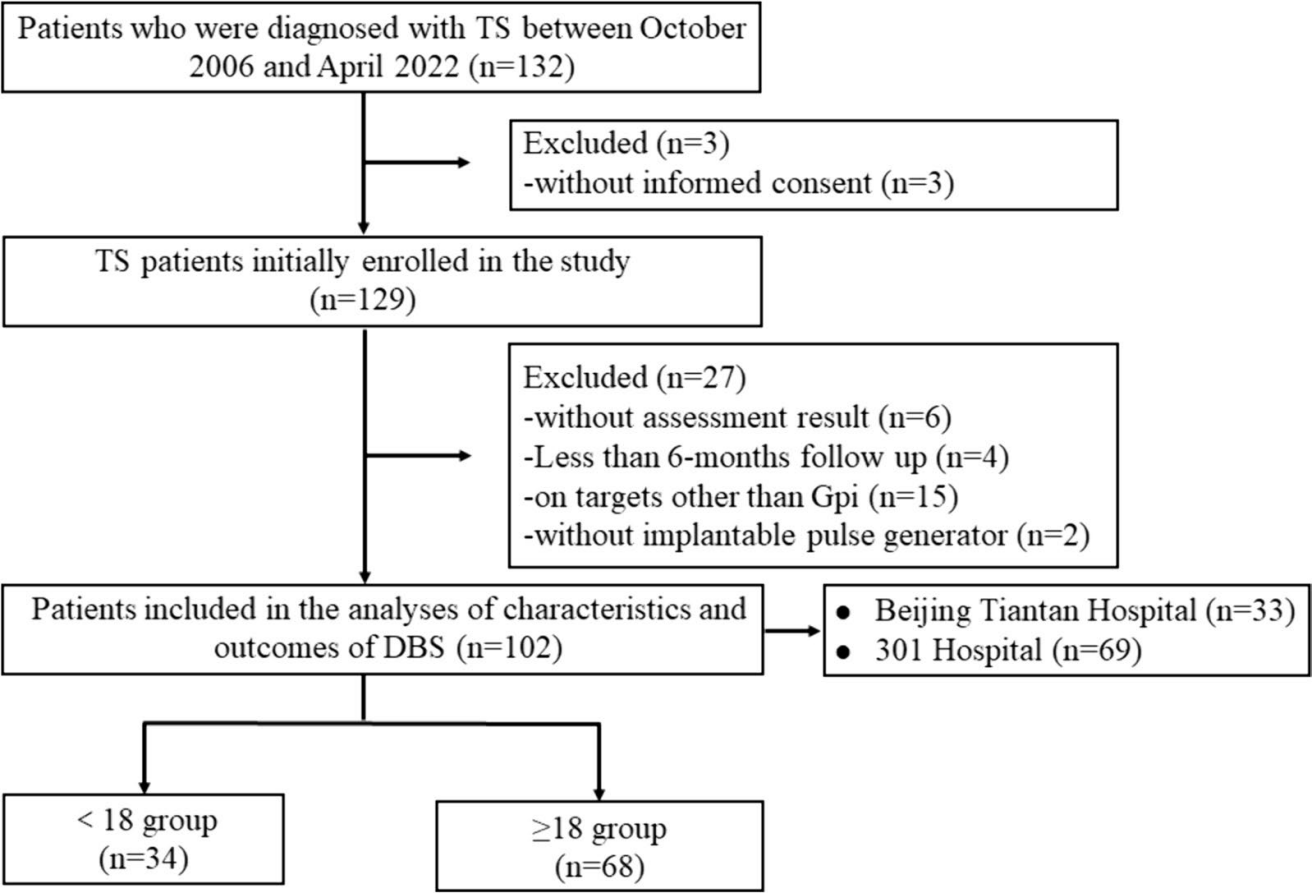
Flow diagram of the enrollment procedure of patients with GTS

Figure 1 shows the enrollment procedure. Before surgery, all patients were confirmed for surgical criteria by experienced specialists in movement and psychiatric disorders and functional neurosurgeries, which included the following: (i) tics should be defined as harmful or malignant for a minimum period of 6 months or should be scored as ≥ 35 on the YGTSS-TTS for a minimal period of 12 months, (ii) the patient should report at least moderate impairment on the YGTSS impairment score or high impairment on the Gilles de la Tourette Quality of Life Scale (GTS-QoL) for a minimal period of 12 months, (iii) patients should be treatment-resistant defined as no response to behavioral and/or pharmacological interventions, and (iv) co-existing psychiatric conditions should be stable for a minimal period of 6 months before surgery and primary impairment of quality of life should be caused by tics [21]. Patients were divided into age at surgery < 18 years (children group) and age at surgery ≥ 18 years (adults group) as the study groups.

### Surgical procedure

DBS implantation was performed under local anesthesia using the Leksell stereotactic system (Elekta Instrument AB, Stockholm, Sweden). After the initial localization of the targets by the SurgiPlan system (Elekta Instrument AB, Stockholm, Sweden), the optimal position was further determined through intraoperative electrophysiological recording and intraoperative test stimulation. All patients were implanted with quadripolar leads (3387 Medtronic, Minneapolis, MN, or L302, Pins Medical, Beijing, China) in bilateral hemispheres. The coordinates of the GPi target were located as its posteroventrolateral parts, which was used in all patients, and the lowest contact was 18–21 mm lateral to the AC-PC, 3 mm anterior to the MCP, and 4–6 mm below the inter-commissural line. The leads were then attached to an implantable pulse generator (IPG) implanted under the clavicle under general anesthesia. Then, the patient underwent a postoperative CT examination for examinations of intracranial hemorrhage and other complications. Finally, the position of the DBS leads was verified by fusing the postoperative CT with the preoperative MR images [22].

The IPG was switched on at the 1-month follow-up after the surgery. Each patient was adjusted with stimulation parameters and medications until reaching an optimal balance of symptom control and minimum adverse effects. Most of the switch-on parameters of DBS were set to a pulse width of 60 µs and a frequency of 130 Hz at switch-on, and the voltage was adjusted according to the patient’s response, increasing the stimulation voltage step by step to reduce stimulation-induced dyskinesia and adverse reactions in behavior.

### Outcome measures

The Yale Global Tic Severity Scale (YGTSS) was assessed as the primary outcome for tic symptoms. The YGTSS was used to assess symptoms such as motor and phonic tics of GTS. The items of the YGTSS scale were summarized into the three domains of motor tics, phonic tics, and an impairment rating, which ranged from a possible score of 0 (no tics) to a maximum score of 100 (extreme severity) [23]. Tic severity was considered according to the most intense tic. The Yale-Brown Obsessive–Compulsive Scale (YBOCS), Beck Depression Inventory (BDI), and Gilles de la Tourette Syndrome-Quality of Life Scale (GTS-QOL) were assessed for symptoms of OCD, depression, and quality of life, respectively [23, 24], which were the secondary outcomes. Scores of the YBOCS [24] and BDI were classified based on Chinese consensus, including none, mild, moderate, and severe.

Patients with GTS were assessed longitudinally at baseline (preoperative), 6 months and 12 months after the surgery, and every 12 months thereafter. The clinical improvement was calculated as ([(preoperative scores − postoperative scores)/prescores] × 100%).

### Statistical analysis

The mean (standard deviation, SD), median with range, or number with percentages were reported for description. The analysis of differences between two samples was performed using the independent/paired* t* test or the rank-sum test (Mann‒Whitney or Wilcoxon test) for continuous variables or *χ*^2^ test (Pearson’s or Fisher’s exact) for categorical variables, as appropriate.

For longitudinal continuous variables, one-way repeated-measures ANOVA was applied to evaluate outcomes assessed at different follow-up periods (7 levels: baseline, at 6 months, at 12 months, at 24 months, at 36 months, at 48 months, at ≥ 60 months and more) within groups. In analyzing differences in outcomes of different age groups (children vs adult groups) and different YGTSS subscores (motor and phonics), multivariate repeated-measures ANOVAs were performed. Post hoc comparisons for pairwise comparisons of different time periods were conducted with Scheffe’s correction. For longitudinal categorical variables, Kaplan‒Meier curves were plotted with log-rank tests for comparisons. All data were analyzed using SPSS software (version 27; IBM; Chicago, IL) and R with packages (version 4.2; R Foundation; Vienna, Austria). All tests were two-tailed, and *p* < 0.05 was considered statistically significant.

## Results

### Patient characteristics

The demographic characteristics of the patients are summarized in Table 1. In total, 102 participants (34 children and 68 adults) were enrolled; 33 of them were enrolled from Beijing Tiantan Hospital, and the other 69 patients were enrolled from the First Medical Center of Chinese PLA General Hospital. As GTS is more likely to occur in males, most (92.2%) of the included patients were men, and 7.8% were women. These patients had a mean age of symptom onset of 9.7 years, and the mean age at surgery was 21.8 years (range 10.0–41.0). The most common psychiatric comorbidities were OCB [76/102 (74.5%)], attention deficit disorder (ADD) [45/102 (44.1%)], depression [83/102 (81.4%)], anxiety [61/102 (59.8%)], and self-injurious behavior (SIB) [7/102 (6.9%)]. The follow-up duration of all patients ranged from 6 to 126 months, with a median follow-up period of 60 months; there was no significant difference between children and adults (*p* = 0.44). Apart from age at surgery, no significant difference was found in sex; age at onset; comorbidities; baseline assessments by YGTSS, YBOCS, BDI, and GTS-QOL; IPG; or requirement centers (all *p* > 0.05), suggesting good comparability (Additional file 1: Table S1).

**Table 1:** Characteristics of all patients with GTS

	Number/total number with data (%), mean ± standard deviation SD, or median (range)			
Characteristics	All patients	Children (*n* = 34)	Adult (*n* = 68)	*p* value
Sex				
Female	8/102 (7.8%)	4/34 (11.8%)	4/68 (5.9%)	0.44
Male	94/102 (92.2%)	30/34 (88.2%)	64/68 (94.1%)	
Ages, years				
Age at onset	9.7 ± 4.3	8.2 ± 3.2	10.4 ± 4.6	0.22
Age at surgery	21.8 ± 6.3	15.9 ± 1.9	24.8 ± 5.6	< 0.001
Comorbidities				
OCB	76/102 (74.5%)	25/34 (73.5%)	51/68 (75.0%)	0.97
ADHD	45/102 (44.1%)	14/34 (41.2%)	31/68 (45.6%)	
Depression	83/102 (81.4%)	23/34 (67.6%)	60/68 (88.2%)	
Anxiety	61/102 (59.8%)	19/34 (55.9%)	42/68 (61.8%)	
SIB	7/102 (6.9%)	3/34 (8.8%)	4/68 (5.9%)	
Assessments				
YGTSS total score	73.2 ± 16.3	72.2 ± 15.2	73.7 ± 16.8	0.65
YGTSS motor score	20.2 ± 3.6	20.3 ± 3.5	20.2 ± 3.6	0.93
YGTSS phonic score	16.7 ± 5.9	16.9 ± 5.4	16.5 ± 6.2	0.996
YGTSS impairment score	35.8 ± 11.1	34.7 ± 10.9	36.3 ± 11.1	0.52
YBOCS total score	18.2 ± 8.5	17.9 ± 9.2	18.3 ± 8.2	0.85
BDI total score	21.4 ± 12.1	22.1 ± 13.2	21.2 ± 11.6	0.85
GTS-QOL total score	72.8 ± 15.6	72.2 ± 14.9	73.1 ± 15.9	0.86
Centers				
Beijing Tiantan Hospital	33/102 (32.4%)	12/34 (35.3%)	21/68 (30.9%)	0.65
Chinese PLA General Hospital	69/102 (67.6%)	22/34 (64.7%)	47/68 (69.1%)	
IPG				
PINS G102/R	38/102 (37.3%)	11/34 (32.4%)	27/68 (39.7%)	0.47
Medtronic Activa PC/RC	64/102 (62.7%)	23/34 (67.6%)	41/68 (60.3%)	
Duration of follow-up, months	60 (6.0–126.0)	60 (12–126)	60 (6–124)	0.98

*Abbreviations: OCB* Obsessive–compulsive behavior, *ADD* Attention-deficit disorder, *SIB* Self-injurious behavior, *YGTSS* Yale Global Tic Severity Scale, *YBOCS* Yale-Brown Obsessive–Compulsive Scale, *GTS-QOL* Gilles de la Tourette Syndrome-Quality of Life Scale, *BDI* Beck Depression Inventory, *IPG* Implantable pulse generator

### Assessments and outcomes

**Fig. 2:**
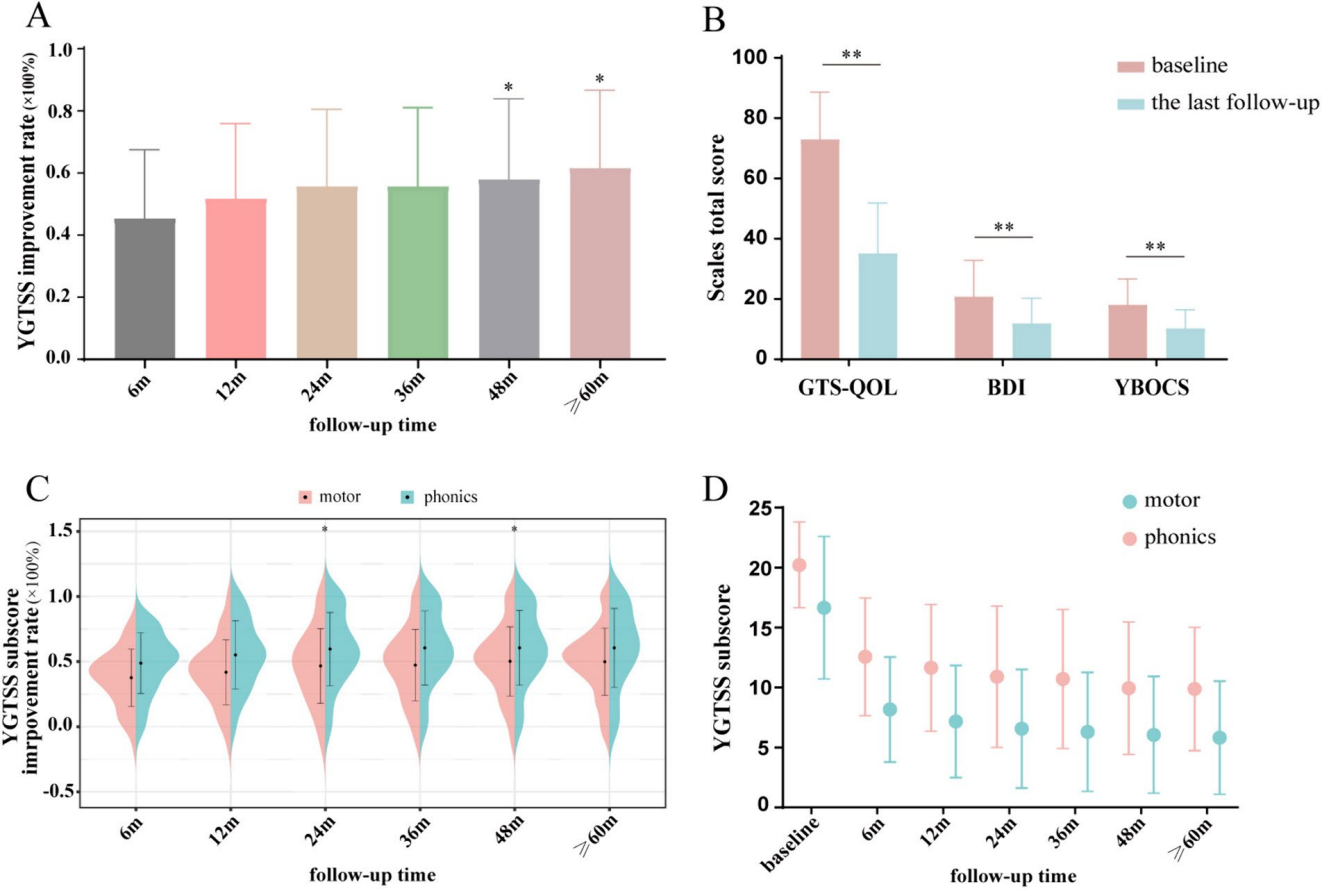
The primary and secondary outcomes in 102 patients. **A** The improvement rate of YGTSS scores at different follow-up time points. **B** The baseline and last follow-up time scores of the GTS-QOL, BDI, and YBOCS. **C** The improvement rate of motor tic and phonic tic scores at different follow-up time points. **D** The baseline and different follow-up time point scores of motor tics and phonic tics. Abbreviations: YGTSS, Yale Global Tic Severity Scale; YBOCS, Yale-Brown Obsessive–Compulsive Scale; BDI, Beck Depression Inventory; GTS-QOL, Gilles de la Tourette Syndrome-Quality of Life Scale; *p < 0.05, **p < 0.01

Figure 2A summarizes the improvements in YGTSS scores. The mean (SD) YGTSS total score was 73.2 (16.3) at baseline and 32.3 (19.4) at the last follow-up, with a mean reduction of 55.3% (24.9%; significantly improved, *p* < 0.001). The mean YGTSS total score significantly improved 45.2%, 51.6%, 55.5%, 55.6%, 57.8%, and 58.1% at 6, 12, 24, 36, 48, and ≥ 60 months of follow-up compared with baseline (all *p* < 0.05), which suggested further improvements with time (Fig. 2A). Greater than 30% improvement was observed in 85.3% of patients, and improvement greater than 60% was noted in 42.2% of patients. Figure 2C and D show the analysis revealing the outcome on the clinical motor tic scores and phonic tic scores. The mean motor tic scores improved 37.6%, 41.7%, 46.2%, 47.2%, 50.1%, and 49.8% at 6, 12, 24, 36, 48, and ≥ 60 months of follow-up, respectively, compared with baseline (all *p* < 0.05). The mean phonic tic scores improved by 48.7%, 55.0%, 59.6%, 60.4%, 60.4%, and 60.4% at 6, 12, 24, 36, 48, and ≥ 60 months of follow-up, respectively, compared with baseline (all *p* < 0.05). As shown in Fig. 2C, there was a significant difference between motor and phonic tic improvements at the 24-month and 48-month follow-up times (59.6% vs 46.2% for 24 months, 60.4% vs 50.1% for 48 months; all *p* < 0.05). YGTSS total scores for different follow-up time points are represented in Tables 2 and 3. Figure 2B summarizes the YBOCS, BDI, and GTS-QOL scores at baseline and at the last follow-up, which suggested significant improvements of 42.0%, 46.3%, and 42.0%, respectively (all *p* < 0.01).

**Table 2:** YGTSS scores in all patients and subgroups at different follow-ups

	All GTS patients	Children	Adult	*P*_*C*_-value
Baseline (*n* = 102)	73.2 ± 16.3	72.2 ± 15.2	73.7 ± 16.8	0.667
6 months (*n* = 102)	39.5 ± 17.1	33.8 ± 16.1	42.3 ± 17.0	0.256
Improvement (%)	45.2%	52.9%	41.4%	
*P*_*B*_-value	< 0.001**	< 0.001**	< 0.001**	
12 months (*n* = 99)	34.7 ± 18.6	29.7 ± 18.2	37.3 ± 18.3	0.382
Improvement (%)	51.6%	58.8%	47.8%	
*P*_*B*_-value	< 0.001**	< 0.001**	< 0.001**	
24 months (*n* = 93)	32.3 ± 19.4	26.9 ± 18.7	35.1 ± 19.2	0.206
Improvement (%)	55.5%	62.8%	51.8%	
*P*_*B*_-value	< 0.001**	< 0.001**	< 0.001**	
36 months (*n* = 84)	32.0 ± 19.7	26.2 ± 19.0	35.3 ± 19.3	0.207
Improvement (%)	55.6%	63.9%	51.0%	
*P*_*B*_-value	< 0.001**	< 0.001**	< 0.001**	
48 months (*n* = 73)	29.9 ± 19.5	23.5 ± 17.9	33.7 ± 19.4	0.152
Improvement (%)	57.8%	66.9%	52.5%	
*P*_*B*_-value	< 0.001**	< 0.001**	< 0.001**	
≥ 60 months (*n* = 54)	26.8 ± 17.8	20.5 ± 14.1	30.8 ± 18.8	0.039*
Improvement (%)	58.1%	61.4%	55.9%	
*P*_*B*_-value	< 0.001**	< 0.001**	< 0.001**	

*P*_*B*_*-value* *p* value for within-group comparison between follow-ups and baseline, *P*_*C*_*-value* *p* value for comparison between the children and adults group, *YGTSS* Yale Global Tic Severity Scale

*p < 0.05, **p < 0.01

**Table 3:** YBOCS, BDI, and GTS-QOL scores in all patients and subgroups at baseline and the last follow-up

	All GTS patients	Children	Adult	*P*_*C*_-value
**YBOCS**				
Pre	18.2 ± 8.5	17.9 ± 9.2	18.3 ± 8.2	0.848
Post	10.2 ± 6.2	9.5 ± 6.7	10.6 ± 5.9	0.280
Improvement (%)	42.0%	45.4%	40.3%	0.049
* P*_*B*_-value	< 0.001**	< 0.001**	< 0.001**	
Follow-up periods	6–126	12–126	6–124	
**BDI**				
Pre	21.4 ± 12.1	22.1 ± 13.2	21.2 ± 11.6	0.846
Post	11.7 ± 8.5	11.7 ± 9.7	11.7 ± 8.0	0.679
Improvement (%)	46.3%	48.9%	45.4%	0.537
* P*_*B*_-value	< 0.001**	< 0.001**	< 0.001**	
Follow-up periods	6–126	12–126	6–124	
**GTS-QOL**				
Pre	72.8 ± 15.6	72.2 ± 14.9	73.1 ± 15.9	0.864
Post	36.2 ± 16.4	32.5 ± 17.5	38.1 ± 15.5	0.092
Improvement (%)	50.6%	55.9%	47.9%	0.359
* P*_*B*_-value	< 0.001**	< 0.001**	< 0.001**	
Follow-up periods	9–126	12–126	9–113	

*P*_*B*_*-value* *p* value for within-group comparison between follow-ups and baseline, *P*_*C*_*-value* *p* value for comparison between the children and adults group, *YBOCS* Yale-Brown Obsessive–Compulsive Scale, *GTS-QOL* Gilles de la Tourette Syndrome-Quality of Life Scale, *BDI* Beck Depression Inventory

*p < 0.05, **p < 0.01

### Comparisons of age categories

**Fig. 3:**
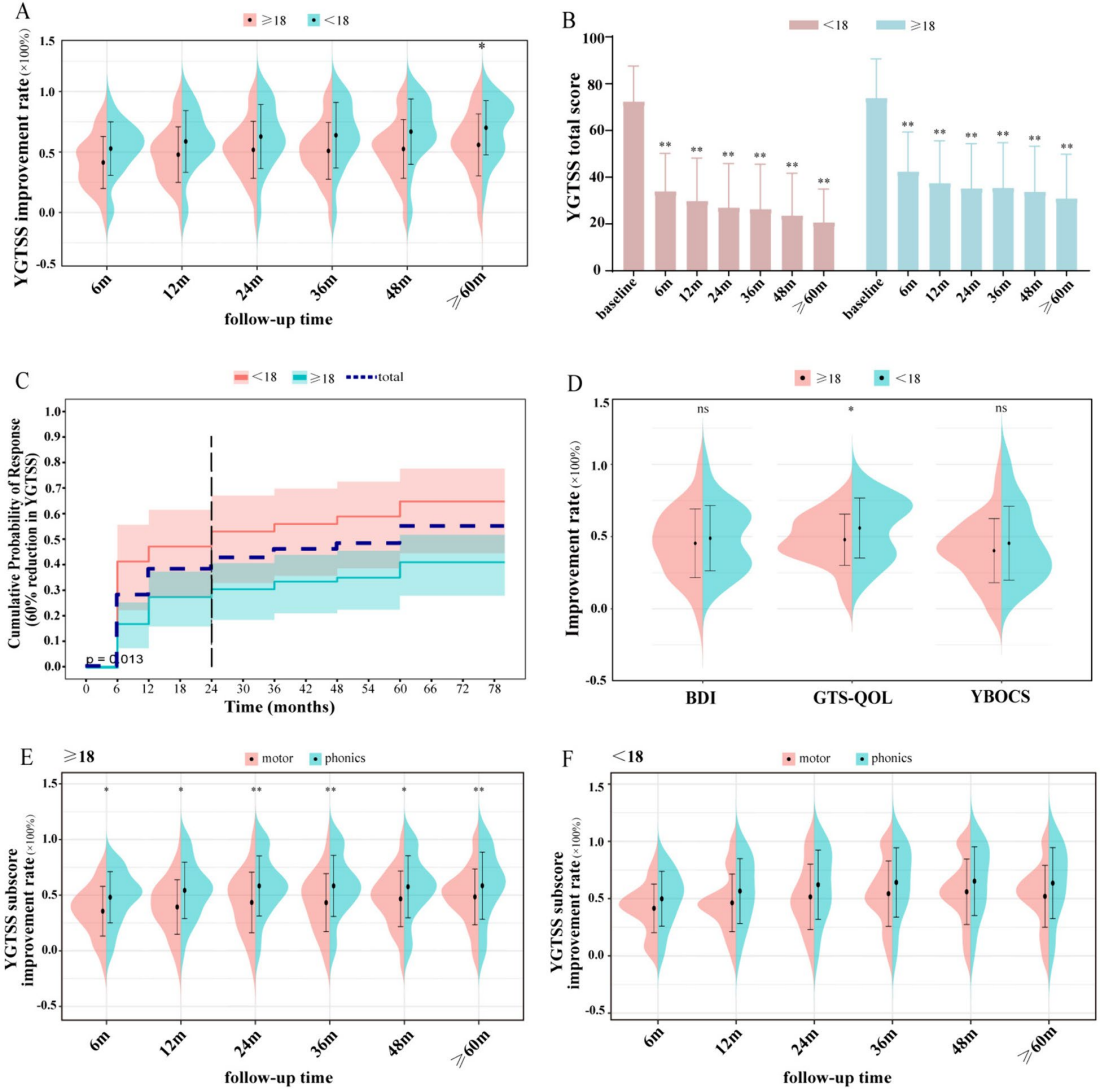
The difference in primary and secondary outcomes between different age categories. **A** The improvement rate of YGTSS scores at different follow-up time points between the child group and adult group. **B** The baseline and different follow-up time point YGTSS scores between the child group and adult group. **C** Survival analysis of 60% reduction in YGTSS scores between the child group and the adult group. **D** The baseline and last follow-up time scores of the GTS-QOL, BDI, and YBOCS between the child group and adult group. **E** The improvement rate of motor tic and phonic tic scores at different follow-up time points in the adult group. **F** The improvement rate of motor tic and phonic tic scores at different follow-up time points in the pediatric group. Abbreviations: YGTSS, Yale Global Tic Severity Scale; YBOCS, Yale-Brown Obsessive–Compulsive Scale; BDI, Beck Depression Inventory; GTS-QOL, Gilles de la Tourette Syndrome-Quality of Life Scale. *p < 0.05; **p < 0.01

Improvements in the YGTSS in comparing children and adults are represented in Fig. 3A. In the pediatric group, the mean YGTSS scores significantly improved by 52.9%, 58.8%, 62.8%, 63.9%, 66.9%, and 61.4% at 6, 12, 24, 36, 48, and ≥ 60 months of follow-up, respectively, compared with baseline (all *p* < 0.05). In the adult group, the mean YGTSS scores significantly improved by 41.4%, 47.8%, 51.8%, 51.0%, 52.5%, and 55.9% at 6, 12, 24, 36, 48, and ≥ 60 months of follow-up, respectively, compared with baseline (all *p* < 0.05). As shown in Fig. 3A, there was a significant difference between children and adults at the ≥ 60-month follow-up (70.1% vs 55.9%, *p* = 0.043), and the same trend was observed at other follow-up time points, but there was no significant difference (all *p* > 0.05). Specific YGTSS scores are summarized in Table 2. In Fig. 3B, comparisons in YGTSS scores of the baseline follow-ups within groups showed significant improvements in the children group and adults group (all *p* > 0.01). Furthermore, survival analysis of the 60% reduction in YGTSS scores between the child group and adult group is presented in Fig. 3C, which shows that the 60% improvement rate was achieved faster in the child group than in the adult group (*p* = 0.013). Figure 3D summarizes improvements in YBOCS, BDI, and GTS-QOL scores between children and adults at the last follow-up. In the child group, the mean improvement rate was 45.4% in the YBOCS, 48.9% in the BDI, and 55.9% in the GTS-QOL. In the adult group, the mean improvement rate was 40.3% in the YBOCS, 45.4% in the BDI, and 47.9% in the GTS-QOL. The improvement rate between these two groups of GTS-QOL had a significant difference, which showed a better improvement in the children group (55.9% vs. 47.9%, *p* = 0.049), while other outcomes did not show significance (all *p* > 0.05). Figure 3E and F show the YGTSS subscore improvement rates in the adult group and child group. This result suggested a significantly higher improvement in phonic tics than in motor tics in adults but not in children.

### Comorbidities and side effects

**Fig. 4:**
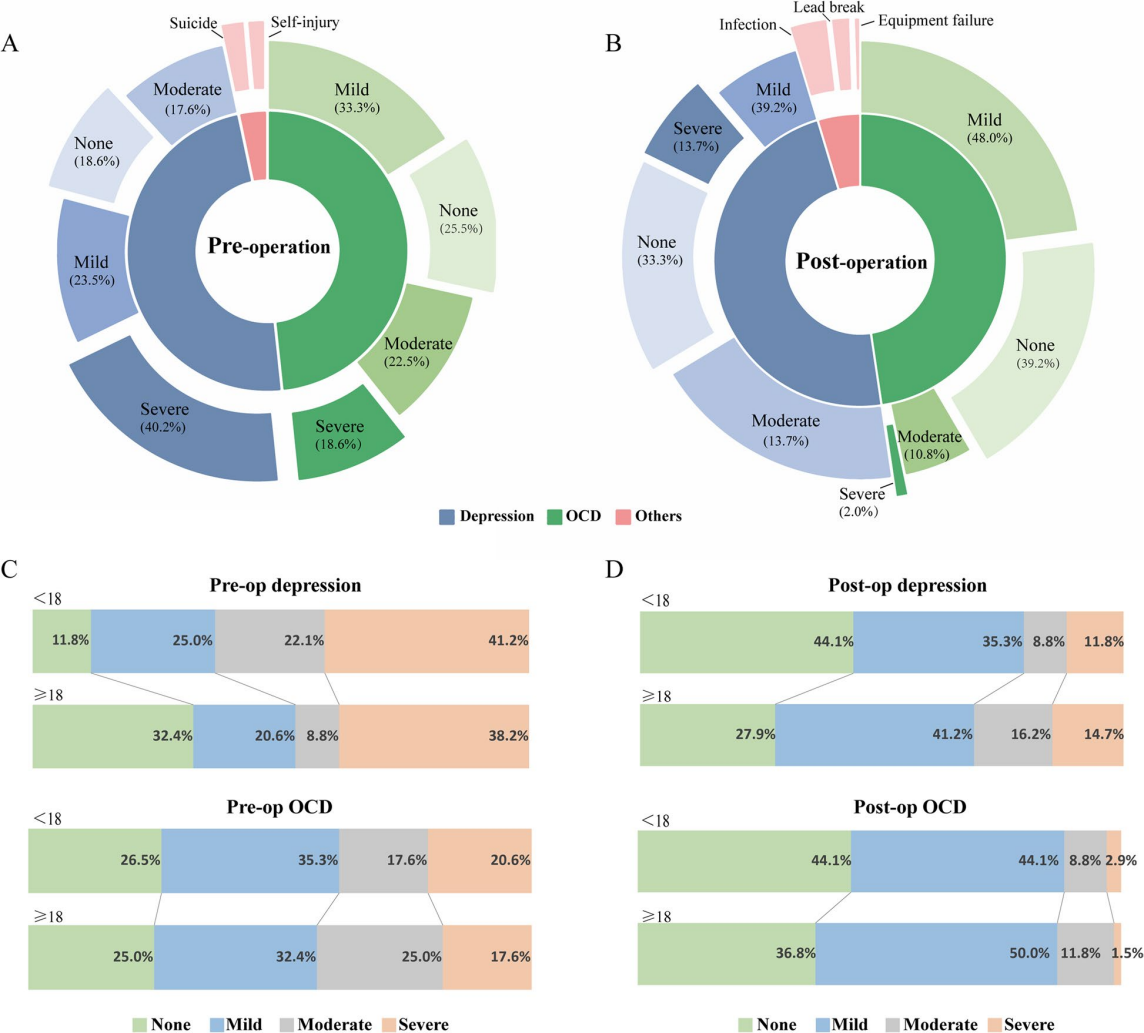
Comorbidities and side effects preoperatively and postoperatively in the pediatric group and adult group.** A** Comorbidities preoperatively in all GTS patients. **B** Comorbidities and postoperative side effects in all GTS patients. **C**, **D** The OCD and depression psychiatric comorbidities preoperatively and postoperatively between subgroups. Abbreviation: OCD, obsessive–compulsive disorder

The comparison between the pre- and postoperative periods regarding depression, OCD, and other comorbidities is shown in Fig. 4. The percentage of patients who were severely depressed preoperatively was 40.2%, and the percentage of patients who were not depressed was 18.6%. However, postoperative severe depression was found in 13.7% of patients, while no symptoms of depression were found in 33.3% of patients. Similarly, preoperative severe OCD was found in 18.6% of patients, and no symptoms of OCD were found in 25.5% of patients, while postoperative severe OCD was found in 2% of patients, and no symptoms of OCD were found in 39.2% of patients, as shown in Fig. 4A and B. In Fig. 4C and D, the percentages of preoperative patients with severe depression in the child and adult groups were 41.2% and 38.2%, respectively, while the percentages of postoperative patients were 11.8% and 14.7%. Similarly, the proportions of patients without preoperative depression were 11.8% and 32.4% in the child and adult groups, respectively, while the proportions of patients without postoperative depression were 44.1% and 27.9%. The average voltage, pulse width, and frequency of the left (right) hemisphere of this cohort of patients at the time of switch-on were 2.3 v (2.4 v), 60 µs (60 µs), and 130 Hz (130 Hz), respectively. The mean voltage, pulse width, and frequency of the left (right) brain hemisphere at the last follow-up were 3.1 v (3.2 v), 88 µs (90 µs), and 169 Hz (169 Hz), respectively. Among the 102 patients, 4 had nausea or vertigo symptoms, 3 had lethargy symptoms, and 1 had headache symptoms after the program control. These symptoms disappeared after parameter adjustment.

## Discussion

GTS is a neurological disorder with motor and phonic tics as the main symptoms, usually with onset in childhood [1, 2, 25]. It is often combined with behavioral disorders such as OCD/OCB, ADHD, SIB, anxiety, and depression [25]. Despite the many treatment options available, there are still patients with severe tic symptoms who are not sensitive to conventional treatment without clinical outcomes [26]. For these inherently refractory GTS patients, DBS can offer a valuable alternative treatment option [27–30].

DBS is an effective treatment for tics and psychiatric comorbidities of GTS patients with a moderate safety profile. In this retrospective clinical study, 102 patients with GTS underwent DBS performed by our team. The mean YGTSS total score significantly improved 45.2%, 51.6%, 55.5%, 55.6%, 57.8%, and 58.1% at 6, 12, 24, 36, 48, and ≥ 60 months of follow-up compared with baseline (all *p* < 0.05), which suggested further improvements with follow-ups. Regarding psychiatric comorbidities and life quality of patients, the mean YBOCS score significantly improved by 42.0% at the last follow-up time, 46.3% in the BDI improvement rate, and 42.0% in the GTS-QOL improvement rate (all *p* < 0.01). Similarly, a meta-analysis studied by Baldermann et al. included 57 studies, which showed that tic symptoms significantly improved over time, with an improvement of 52.7% on the YGTSS (*p* < 0.01) [27]. Another two randomized controlled trials showed a significant improvement of 40.1% and 69.5% with GPi-DBS at the end of the open-label phase after 6–48 months, respectively [18, 29]. In addition, Baldermann et al*.* also found a median improvement of OCD of 31.3% on the YBOCS and mood of 38.9% on the BDI [27]. Recently, Shu Wang et al. further evaluated the efficacy and safety of combined deep brain stimulation with capsulotomy for comorbid motor and psychiatric symptoms in Tourette’s syndrome by reviewing clinical data and meta-analysis [31].

DBS is still controversial in the treatment of GTS for several reasons, for example, the fact that tic symptoms may improve in adolescence even without treatment for some GTS patients [25, 32, 33]. Many studies have shown that tic symptoms decrease significantly during adolescence in approximately 40% of patients with GTS, another 40% may disappear completely, and the remaining 20% have tic symptoms that remain pronounced or even tend to worsen after adolescence and require long-term treatment [34, 35]. Therefore, the Tourette Syndrome Association (TSA), in its first guidelines for GTS treated by DBS issued in 2006, stated that no patient under the age of 25 years would be allowed to undergo DBS surgery [20]. The reason for this was that it could not ensure that the leads and IPG might be implanted in a subset of GTS patients whose symptoms might have disappeared on their own during adolescence. However, the TSA’s latest consensus guidelines, which were issued in 2015, have changed the age limitation for patients undergoing surgery and moved away from the previously recommended age limit of “patients must be older than 25 years” [7]. These guidelines state that patients under the age of 18 years old should be operated on in consultation with their local ethics committee or institutional review board, which relaxed the age requirement. In this retrospective clinical study, all patients were divided into two groups based on the age of 18. Among the different age subgroups, there were significant differences in the improvement of the YGTSS improvement rate at ≥ 60 follow-up times. This result suggested that there were better surgical results in the child group than in the adult group in terms of the long-term efficacy of DBS for GTS, which also does not exclude the possibility of receiving a patient’s self-healing. In addition, there was a smaller proportion of total depression in the child group than in the adult group at baseline, which could indirectly indicate that the comparison of the improvement in tic symptoms is relatively less influenced by the confounding factor of depressive mood. There was also a significant difference between motor tic and phonic tic improvement in the adult group, which indicated that DBS was more effective in treating phonic tics than motor tics. A similar trend was evident in the children group, while no significant differences emerged, possibly due to the relatively small sample size. In addition, the improvement in GTS-QOL was significantly better in the pediatric group than in the adult group, suggesting that DBS would provide a better improvement in quality of life for pediatric patients.

Regarding the possibility of self-healing, we do not exclude the existence of such a trend. However, it is true that the symptoms of the GTS patients included in this study were refractory and that the patients had heavy tic symptoms. These patients were not observed to have a significant tendency to self-heal, so the greater likelihood of symptom improvement should be attributed to DBS, but how to quantify this is something that can only be achieved by conducting randomized controlled trials (RCTs). However, we have provided the important value that DBS can be performed in refractory, heavily symptomatic children with even better results than in adults. Therefore, we recommend that the age limit for surgery in patients with GTS can be relaxed appropriately by the professional assessment and willingness of patients and their families so that patients can be treated earlier when their symptoms have begun to affect their quality of life. A growing number of studies show that even though tic symptoms resolve in adolescence, the symptoms of GTS patients can still seriously affect psychological development and can even jeopardize their educational and work opportunities, social interactions, and relationships, which will cause irreversible damage [7]. Another reason to consider DBS surgery early in the course of the disease is that GTS patients are at a high risk of self-inflicted tics, including violent cervical tics, which may cause cervical spondylitis myelopathy and secondary neurological dysfunction [36]. This group of patients is collectively referred to as “malignant” GTS, and approximately 5% of GTS patients will develop this type [37]. Delaying surgery for these patients may not only cause permanent damage to their cognitive, emotional, and social development but may also be life-threatening [38]. This is what we often say: “patient choice should optimize the individual risk–benefit ratio” [39]. In addition, studies have shown that DBS is a safe surgical operation with a less than 1% risk of serious complications, including bleeding and neurological dysfunction [7, 27, 40]. However, some studies have also suggested that GTS is a chronic disease and that surgical treatment is not urgent [41, 42]. We believe that the greatest impediment to our current exploration of GTS is the impossibility of predicting the natural course of tic symptom improvement in GTS, so we cannot use traditional chronic conditions to recommend DBS for GTS [43]. These patients choose to undergo surgery early in the course of their illness not because it is a chronic condition but because they cannot tolerate the actual pain caused by the disease. Postponing surgery at this time can only lead to a deterioration in quality of life and the destruction of physical and mental health during this important period of life. Even if the treatment for DBS remains effective for several years after adolescence, waiting too long to undergo surgery can lead to irreparable personal and social harm to the patient. In 2016, Zekaj et al. described a patient who underwent DBS treatment early, whose tics decreased significantly in severity during adolescence [44]. The symptoms were still well improved when the IPG was eventually turned off in this patient [44]. Whether DBS treatment can be considered a temporary therapeutic application needs to be confirmed by further clinical studies. It is therefore impossible to determine whether the improved results in the child cohort are a result of DBS treatment. However, the fact that they show similar or possibly better symptom relief is an indication that the treatment is effective and safe even in child patients and that we will follow up with RCTs to further shed more light on this issue.

There are several limitations to this study. First, the retrospective nature of this analysis has several inherent limitations. Not all variables can be fully controlled. Second, current evidence on the optimal age at surgery or decision of DBS in children with GTS is still not enough, and evidence from randomized controlled trials is needed. Additionally, due to the characteristics of available GTS patients, more patients in our cohorts were males, which might introduce selection bias.

## Conclusions

The present study supports that the long-term efficacy and safety of DBS are acceptable for both children and adults with GTS, and a higher improvement is observed in phonic tics than in motor tics. Surgeries performed for patients younger than 18 years seem to show acceptable long-term efficacy and safety and were not associated with increased risks of loss of benefit compared to patients older than 18 at the time of surgery. These findings could be helpful in evaluating efficacy for children and adults and might be helpful in the decision of optimal age of DBS for GTS patients. However, earlier surgical ages should also be cautious to ensure treatment refractoriness and safety.

### Supplementary Information

Supplementary Material 1. Tables S1. Table S1-The clinical scores in all patients and different IPG subgroups (PINS G102/R group or Medtronic Activa PC/RC group) at different follow-ups.

## Data Availability

The datasets used and/or analyzed during the current study are available from the corresponding author upon reasonable request.

## Abbreviations

ADD: Attention-deficit disorder
BDI: Beck Depression Inventory
DBS: Deep brain stimulation
GTS: Gilles de la Tourette syndrome
GTS-QOL: Gilles de la Tourette Syndrome-Quality of Life Scale
OCD: Obsessive-compulsive disorder
SIB: Self-injurious behavior
YBOCS: Yale-Brown Obsessive–Compulsive Scale
YGTSS: Yale Global Tic Severity Scale

## References

[1] Müller-Vahl KR, Szejko N, Saryyeva A (2021). Randomized double-blind sham-controlled trial of thalamic versus GPi stimulation in patients with severe medically refractory Gilles de la Tourette syndrome. Brain Stimul.

[2] Robertson MM (2000). Tourette syndrome, associated conditions and the complexities of treatment. Brain.

[3] Leckman JF (2002). Tourette's syndrome. Lancet.

[4] Servello D, Galbiati TF, Balestrino R (2020). Deep brain stimulation for Gilles de la Tourette syndrome: toward limbic targets. Brain Sci.

[5] Peterson BS (2001). Neuroimaging studies of Tourette syndrome: a decade of progress. Adv Neurol.

[6] Wang Z, Maia TV, Marsh R, Colibazzi T, Gerber A, Peterson BS (2011). The neural circuits that generate tics in Tourette's syndrome. Am J Psychiatry.

[7] Schrock LE, Mink JW, Woods DW (2015). Tourette syndrome deep brain stimulation: a review and updated recommendations. Mov Disord.

[8] Fraint A, Pal G (2015). Deep brain stimulation in Tourette's syndrome. Front Neurol.

[9] Neumann WJ, Huebl J, Brücke C (2018). Pallidal and thalamic neural oscillatory patterns in Tourette's syndrome. Ann Neurol.

[10] Kenney C, Simpson R, Hunter C (2007). Short-term and long-term safety of deep brain stimulation in the treatment of movement disorders. J Neurosurg.

[11] Babel TB, Warnke PC, Ostertag CB (2001). Immediate and long term outcome after infrathalamic and thalamic lesioning for intractable Tourette's syndrome. J Neurol Neurosurg Psychiatry.

[12] Meng F, Hu W, Wang S (2023). Utilization, surgical populations, centers, coverages, regional balance, and their influential factors of deep brain stimulation for Parkinson's disease: a large-scale multicenter cross-sectional study from 1997–2021. Int J Surg.

[13] Wang S, Zhu G, Shi L (2023). Closed-loop adaptive deep brain stimulation in Parkinson's disease: procedures to achieve it and future perspectives. J Parkinsons Dis.

[14] Vandewalle V, van der Linden C, Groenewegen HJ, Caemaert J (1999). Stereotactic treatment of Gilles de la Tourette syndrome by high frequency stimulation of thalamus. Lancet.

[15] Bajwa RJ, de Lotbinière AJ, King RA (2007). Deep brain stimulation in Tourette's syndrome. Mov Disord.

[16] Cannon E, Silburn P, Coyne T, O'Maley K, Crawford JD, Sachdev PS (2012). Deep brain stimulation of anteromedial globus pallidus interna for severe Tourette's syndrome. Am J Psychiatry.

[17] Okun MS, Foote KD, Wu SS (2013). A trial of scheduled deep brain stimulation for Tourette syndrome: moving away from continuous deep brain stimulation paradigms. JAMA Neurol.

[18] Welter ML, Houeto JL, Thobois S (2017). Anterior pallidal deep brain stimulation for Tourette's syndrome: a randomised, double-blind, controlled trial. Lancet Neurol.

[19] Rissardo JP, Vora NM, Tariq I, Mujtaba A, Caprara ALF (2023). Deep brain stimulation for the management of refractory neurological disorders: a comprehensive review. Medicina (Kaunas, Lithuania).

[20] Mink JW, Walkup J, Frey KA (2006). Patient selection and assessment recommendations for deep brain stimulation in Tourette syndrome. Mov Disord.

[21] Martino D, Deeb W, Jimenez-Shahed J (2021). The 5 pillars in Tourette syndrome deep brain stimulation patient selection: present and future. Neurology.

[22] Horn A, Li N, Dembek TA (2019). Lead-DBS v2: Towards a comprehensive pipeline for deep brain stimulation imaging. Neuroimage.

[23] Leckman JF, Riddle MA, Hardin MT (1989). The yale global tic severity scale: initial testing of a clinician-rated scale of tic severity. J Am Acad Child Adolesc Psychiatry.

[24] Goodman WK, Price LH, Rasmussen SA (1989). The yale-brown obsessive compulsive scale. I. Development, use, and reliability. Arch Gen Psychiatry.

[25] Hirschtritt ME, Lee PC, Pauls DL (2015). Lifetime prevalence, age of risk, and genetic relationships of comorbid psychiatric disorders in Tourette syndrome. JAMA Psychiat.

[26] Budman CL (2014). The role of atypical antipsychotics for treatment of Tourette's syndrome: an overview. Drugs.

[27] Baldermann JC, Schüller T, Huys D (2016). Deep brain stimulation for Tourette-syndrome: a systematic review and meta-analysis. Brain Stimul.

[28] Martinez-Ramirez D, Jimenez-Shahed J, Leckman JF (2018). Efficacy and safety of deep brain stimulation in Tourette syndrome: The International Tourette syndrome deep brain stimulation public database and registry. JAMA Neurol.

[29] Kefalopoulou Z, Zrinzo L, Jahanshahi M (2015). Bilateral globus pallidus stimulation for severe Tourette's syndrome: a double-blind, randomised crossover trial. Lancet Neurol.

[30] Dai L, Xu W, Song Y (2022). Subthalamic deep brain stimulation for refractory Gilles de la Tourette's syndrome: clinical outcome and functional connectivity. J Neurol.

[31] Wang S, Fan S, Gan Y (2024). Efficacy and safety of combined deep brain stimulation with capsulotomy for comorbid motor and psychiatric symptoms in Tourette's syndrome: Experience and evidence. Asian J Psychiatr.

[32] Coulombe MA, Elkaim LM, Alotaibi NM (2018). Deep brain stimulation for Gilles de la Tourette syndrome in children and youth: a meta-analysis with individual participant data. J Neurosurg Pediatr.

[33] Grant RA, Halpern CH, Baltuch GH, O'Reardon JP, Caplan A (2014). Ethical considerations in deep brain stimulation for psychiatric illness. J Clin Neurosci.

[34] Bloch MH, Leckman JF (2009). Clinical course of Tourette syndrome. J Psychosom Res.

[35] Robertson MM (2015). A personal 35 year perspective on Gilles de la Tourette syndrome: assessment, investigations, and management. Lancet Psychiatry.

[36] Hauseux PA, Cyprien F, Cif L (2017). Long-term follow-up of pallidal deep brain stimulation in teenagers with refractory Tourette syndrome and comorbid psychiatric disorders: About three cases. Eur J Paediatr Neurol.

[37] Jankovic J, Kurlan R (2011). Tourette syndrome: evolving concepts. Mov Disord.

[38] Xu W, Zhang C, Deeb W (2020). Deep brain stimulation for Tourette's syndrome. Transl Neurodegener.

[39] Clausen J (2010). Ethical brain stimulation - neuroethics of deep brain stimulation in research and clinical practice. Eur J Neurosci.

[40] Akbarian-Tefaghi L, Zrinzo L, Foltynie T (2016). The use of deep brain stimulation in Tourette syndrome. Brain Sci.

[41] Reese HE, Vallejo Z, Rasmussen J, Crowe K, Rosenfield E, Wilhelm S (2015). Mindfulness-based stress reduction for tourette syndrome and chronic tic disorder: a pilot study. J Psychosom Res.

[42] Yin TK, Chiu NT (2004). A computer-aided diagnosis for distinguishing Tourette's syndrome from chronic tic disorder in children by a fuzzy system with a two-step minimization approach. IEEE Trans Biomed Eng.

[43] Müller-Vahl KR, Roessner V (2011). Treatment of tics in patients with tourette syndrome: recommendations according to the European society for the study of tourette syndrome. Mov Disord.

[44] Servello D, Zekaj E, Saleh C, Lange N, Porta M (2016). Deep brain stimulation in Gilles de la Tourette syndrome: what does the future hold? A cohort of 48 Patients. Neurosurgery.

