# Supplementary material for: Comparison of children and adults in deep brain stimulation for Tourette Syndrome: a large-scale multicenter study of 102 cases with long-term follow-up
Source: BMC Med. 2024 May 30;22:218. doi: 10.1186/s12916-024-03432-w (PMC11141040; doi:10.1186/s12916-024-03432-w)
Supplement: Supplementary file 1 — Supplementary Material 1. Tables S1. Table S1-The clinical scores in all patients and different IPG subgroups (PINS G102/R group or Medtronic Activa PC/RC group) at different follow-ups. [file 12916_2024_3432_MOESM1_ESM.docx]

Table S1 The clinical scores in all patients and different IPG subgroups (PINS G102/R group or Medtronic Activa PC/RC group) at different follow-ups.

|  | All GTS patients | PINS G102/R group | Medtronic Activa PC/RC group | *P_C_*-value |
| --- | --- | --- | --- | --- |
| **YGTSS** |  |  |  |  |
| Baseline (n=102) | 73.2±16.3 | 74.9±16.3 | 72.2±16.2 | 0.296 |
| 6-months (n=102) | 39.5±17.1 | 42.0±17.7 | 38.0±16.6 | 0.326 |
| 12-months (n-99) | 34.7±18.6 | 37.6±18.7 | 33.0±18.3 | 0.235 |
| 24-months (n=93) | 32.3±19.4 | 36.5±20.4 | 30.0±18.4 | 0.128 |
| 36-months (n=84) | 32.0±19.7 | 38.1±21.2 | 29.5±18.4 | 0.069 |
| 48-months (n=73) | 29.9±19.5 | 36.8±21.3 | 27.6±118.3 | 0.256 |
| ≥60-months (n=54) | 26.8±17.8 | 25.4±9.1 | 27.1±19.3 | 0.917 |
| **YBOCS** |  |  |  |  |
| Pre | 18.2±8.5 | 16.5±9.3 | 19.1±7.8 | 0.191 |
| Post | 10.2±6.2 | 10.0±7.1 | 10.4±5.5 | 0.270 |
| **BDI** |  |  |  |  |
| Pre | 21.4±12.1 | 19.8±12.5 | 22.4±11.7 | 0.343 |
| Post | 11.7±8.5 | 10.3±8.4 | 12.6±8.4 | 0.150 |
| **GTS-QOL** |  |  |  |  |
| Pre | 72.8±15.6 | 73.0±14.6 | 72.7±16.1 | 0.889 |
| Post | 36.2±16.4 | 36.4±17.3 | 36.1±15.9 | 0.940 |

*P_C_*-value: *P* value for comparison between the different IPG groups. YGTSS, Yale Global Tic Severity Scale; YBOCS, Yale Brown Obsessive Compulsive Scale; GTS-QOL, Gilles de la Tourette Syndrome-Quality of Life Scale; BDI, Beck Depression Inventory.
